# 57. Rheumatology at the interface of medicine: haemphagocytic lymphohistiocytosis/macrophage activation syndrome: an under-recognised syndrome in the acutely unwell patient

**DOI:** 10.1093/rap/rky034.020

**Published:** 2018-09-20

**Authors:** Robert D Sandler, Stuart J Carter, Rachel S Tattersal

**Affiliations:** Rheumatology, Sheffield Teaching Hospitals NHS Foundation Trust, Sheffield, UNITED KINGDOM

**Introduction:** Haemophagocytic lymphohistiocytosis (HLH) is a rare hyperinflammatory syndrome, characterised by the inappropriate survival of histiocytes and cytotoxic T-lymphocytes. HLH is under-recognised but should be suspected in the deteriorating, febrile patient and may present to any medical speciality. Delay in treatment leads rapidly to critical illness and death. Primary HLH results from a genetic defect which usually presents in infancy and is treated by stem cell transplant. Secondary HLH is classically triggered by autoimmune disease, particularly systemic juvenile idiopathic arthritis (sJIA) or systemic lupus eythematosis (SLE) when it is termed macrophage activation syndrome (MAS). It is also triggered by infection (particularly Epstein Barr virus and Varicella Zoster) and malignancy (especially lymphoma). In adults mortality of HLH is 50-75% almost certainly because of late recognition and treatment. Diagnosis requires a high index of suspicion and interpretation of common investigations including full blood count (FBC), lipids, fibrinogen, liver function tests (LFT) and serum ferritin (often highly elevated >10,000 μg/L pathognomic of HLH/MAS in absence of iron overload). Bone marrow biopsy is not required for diagnosis. The H-score can be used to calculate the probability of secondary HLH and in the context of sJIA there are agreed diagnostic MAS criteria. Treatment of HLH involves aggressive immunosuppression, alongside management of the underlying trigger and recent guidance for recognition and treatment of adults with acquired HLH has been published. Due to lack of awareness of HLH among physicians diagnosis may be delayed or even missed leading to poor outcomes, highlighting a potential to improve identification and treatment of HLH and save lives as a result. Rheumatologists have a key part to play in recognition and treatment of HLH/MAS. We present four cases of HLH seen between 2014-2017 highlighting the clinical features, diagnosis and management strategies to promote the rheumatology-general medicine interface.

**Case description:** Case 1: An HLH post-stem cell transplant 55-year-old man, usually fit and well, diagnosed with chronic myeloid leukaemia (CML) underwent allogenic stem cell transplant. Three months later presented with transaminitis, rash and diarrhoea and treated for graft-versus-host disease (GvHD). Three months later, he was admitted with acute confusion, acute kidney injury (AKI) and pancytopenia, requiring intensive therapy unit (ITU) admission. He was treated presumptively for infection although no positive cultures were obtained. Eight months later he presented with fever and rigors and splenomegaly: a bone marrow biopsy showed reactive changes. There were no positive cultures from blood, CSF, urine, throat, skin or Hickmann line. Ferritin was 33 376μg/L, triglycerides 3.7mmol/L, fibrinogen 1.1g/L raising the suspicion of HLH. He was started on dexamethosone and ciclosporin A (CSA) which showed clinical improvement but intermittent pyrexia. He deteriorated twelve days later with type 1 respiratory failure and hypotension, requiring further ITU admission at which point his ferritin was > 100 000μg/L. He was then treated with intravenous (IV) methylprednisolone (MP) and IV Immunoglobulin (IVIg) and also rituximab, as EBV PCR was 54 700 copies/mL, the likely trigger for HLH. Ferritin started to fall but he had worsening AKI and persistent cytopenia so he was given Anakinra, in an attempt to terminate the cytokine storm. There was a swift improvement, and he was restarted on oral prednisolone and anakinra was withdrawn. He deteriorated again with confusion and pyrexia, requiring readmission to ITU. After initially repeating IVMP and IVIG, anakinra was reintroduced with good effect. He was eventually discharged on anakinra and despite CKD and persistently abnormal LFTs, he has remained well.

Case 2: An HLH post varicella zoster infection 74-year-old man with bronchiectasis, but a high functional level, admitted with 10 days of fatigue, lethargy and intermittent confusion. Despite three days of treatment for pneumonia with IV tazocin he became more confused, developed abnormal liver function tests (LFTs) and remained febrile. Despite no positive cultures he was escalated to meropenem, but continued to spike temperatures and developed a pancytopenia, prompting a haematology review and bone marrow biopsy. He was resuscitated after cardiac arrest and taken to ITU. At this point, an ITU consultant queried HLH, with ferritin on 15 554μg/L despite no clear trigger. It then transpired he had shingles three weeks prior to admission and hadn’t been quite right since. He was treated with IVMP and IVIG resulting in rapid resolution of pyrexia and normalisation of ferritin level and full blood count. After discharge from ITU, he made little communicative progress and a CT head demonstrated significant hypoxic brain injury from the cardiac arrest, making chance of recovery slim. He died a week later following a second cardiac arrest.

Case 3: An HLH in sJIA 17-year-old man with sJIA and previous pericarditis, treated with methotrexate (MTX) and tocilizumab (TOC) was admitted with two days of fever and productive cough. He was treated for pneumonia but rapidly deteriorated prompting ITU admission and intubation. He had persistent pyrexia, worsening rash (attributed to his sJIA) and deteriorating LFTs. He had a generalised seizure and was admitted to a specialist neuro ITU. His ferritin was 24 814μg/L, with neutropenia, thombocytopenia and a bone marrow biopsy showing evidence of haemophagocytosis. An MRI brain demonstrated vasogenic oedema, consistent with posterior reversible encephalopathy syndrome (PRES), the aetiology of which presumed to be a combination of HLH and ciclosporin toxicity. He remained neutropenic but well, until deterioration with worsening generalised maculopapular rash. He was treated with IVMP, CSA (until he developed toxicity) and IVIG. Despite this he remained unwell and required etoposide, a powerful chemotherapeutic agent with anti-macrophage effects. He made a complete recovery within a week and stabilised on TOC and MTX.

Case 4: An HLH in SLE 39-year-old Chinese man, living in the UK for 10 years, with no past medical history admitted following a collapse. He also reported recent history of oral ulcers, joint pain, rash, night sweats, weight loss (10kg) and it was noted that he was confused, abbreviated mental test (AMT) 7/10. On examination he was febrile, and had cervical/axillary/iliac lymphadenopathy. Urine dip showed blood and protein, with a urine protein creatinine ratio of 125. Further investigations showed a positive ANA, ENA, RNP, DsDNA and low complement, consistent with systemic lupus erythematosus (SLE). Six days post-admission, he had a rapid deterioration in consciousness with Glasgow coma scale (GCS) of six. Blood tests showed worsening liver function, ferritin 52 000μg/L and raised triglycerides. He was treated for HLH with IVMP and IVIG, resulting in rapid resolution of pyrexia, return to GCS of 15 and progressive improvement in rash, joint pain and proteinuria. He remained stable on a reducing wean of oral prednisolone along with introduction of mycofenylate mofetil (MMF) and hydroxychlorquine (HCQ).

**Discussion:** Secondary HLH is under-recognised in adolescent and adult patients and should be considered in both the rheumatology and general medical population. New guidance suggests that early treatment with immunosuppression (initially methylprednisolone +/- IVIG and early consideration of anakinra) is both safe and effective in the critically unwell patient so raising awareness of HLH is key. Clinicians need to have a high index of suspicion and in particular should think of HLH in patients with fever, confusion, hepatosplenomegaly, dual or pan- lineage cytopenia and multi-organ failure. Serum markers ferritin, triglycerides, fibrinogen and LFTs can be used alongside the clinical features to guide diagnosis. The H-score, a free online calculator, is a validated tool, into which these parameters are inputted to give a probability of HLH. The two rheumatology cases here demonstrate that rheumatologists are aware of HLH and treat early with good outcome. We have presented two general medical cases also with relatively good outcome reflecting the HLH network set up in our Trust resulting in both patients having early recognition of HLH by their non-rheumatology parent teams and early, combined intervention. We recognize that HLH generally and particularly that presenting as a result of lymphoma or other malignancy has a poor outcome. Our HLH network would suggest that HLH is not rare; between 2014 and 2018 we have been involved in managing around 30 cases. Better awareness of HLH in rheumatology and beyond will improve patient care

**Key Learning Points:** The febrile, thrombocytopaenic and unwell/deteriorating with no identified pathogen or disease should mandate estimation of serum ferritin and consideration of HLH. Screen these patients using serum ferritin, and stratify their risk of HLH using the H score Early treatment involving IVMP, IVIG, CSA and Anakinra can help to abort the cytokine storm, but the underlying trigger, such as EBV infection, malignancy or autoimmune disease must be treated concurrently. Rheumatologists should work with other medical specialties to identify patients with HLH and institute early treatment.

**Disclosure: R.D. Sandler:** None. **S.J. Carter:** None. **R.S. Tattersal:** None.

